# Leveraging No-Code Digital Platforms for Designing an Integrated Smartphone-Based Ecological Momentary Intervention With Cognitive Behavioral Therapy for Mental Health Care: Development and Usability Study

**DOI:** 10.2196/77036

**Published:** 2025-11-21

**Authors:** Stephanie Ming Yin Wong, Melody Ho Ching Ip, Jessica Kang Qi Lee, Terry Yat Sang Lum, Ralf Schwarzer

**Affiliations:** 1 Department of Social Work and Social Administration, University of Hong Kong, Rm 702e, The Jockey Club Tower, Centennial Campus, Hong Kong, China (Hong Kong), 852 3917 2288; 2 Department of Psychology, Freie Universität Berlin, Berlin, Germany

**Keywords:** cognitive behavioural therapy, ecological momentary intervention, blended care, mental health care, depression, health behaviour change, health action process model, user-centred design, peer support, mobile apps, digital intervention

## Abstract

**Background:**

The rising burden of disease associated with mental disorders calls for evidence-based psychological interventions that can be swiftly scaled up. Blending smartphone-based mental health apps (MHapps) for delivering ecological momentary interventions (EMIs) with traditional in-person interventions may have the benefits of improving treatment adherence, facilitating the application of learned techniques into everyday life, and, in turn, enhancing clinical response. However, previous work has shown that most existing MHapps were developed for specific research studies or for profit, thereby making them difficult to adapt, particularly in time-limited and resource-constrained settings.

**Objective:**

This study aimed to demonstrate how a person-centered and theory-informed MHapp could be developed in a timely and low-cost manner for use as part of blended care, using a phased approach. Given the scarcity of digital mental health interventions for older adults, we adopted a participatory research approach to co-design the blended intervention with 2 groups of older adults.

**Methods:**

In Phase 1, we reviewed existing MHapps with consideration of whether they could be adapted by individual researchers or clinicians, their key functions, and whether their efficacy had been tested. “No-code” app builders were additionally reviewed, which may be alternatives if no MHapp can be used. In Phase 2, following the IDEAS (Integrate, Design, Assess, and Share) framework, we built a prototype according to users’ needs, with its content informed by theories of cognitive behavioral therapy (CBT) and the Health Action Process Approach. The prototype was then tested and refined over 2 rounds of 3-session co-design workshops with peer supporters (n=8) and service users (n=5) from a stepped-care intervention for older adults with depressive symptoms. Usability testing was conducted with both stakeholder groups in Phase 3.

**Results:**

Of the 149 MHapps identified, only 43 (28.9%) can be publicly downloaded. Four (8.3%) of them can be partially adapted, although no new content can be directly added. We therefore developed the MHapp using m-Path (a spin-off from KU Leuven's Faculty of Psychology), which was the only existing no-code app development platform designed for mental health interventions. A prototype incorporating CBT-based homework and behavior change techniques informed by the Health Action Process Approach was built, with its refined version rated as highly easy to use and acceptable by both stakeholder groups.

**Conclusions:**

By integrating CBT with EMI, we demonstrated the feasibility and acceptability of a novel blended care model for reference in future work. Preliminary findings suggest high usability and clinical relevance, highlighting the potential of leveraging no-code platforms to facilitate scalable, theory-driven interventions that extend mental health support beyond traditional settings. Grounding the blended intervention in evidence-based psychological and health behavior change theories, coupled with user involvement throughout the design process, may improve clinical efficacy and reduce implementation barriers, which are areas for further investigation in future work.

## Introduction

### Background

Mental disorders constitute some of the leading causes of the global burden of disease [1]. Not only do they have implications for the health care system and economy, but they also affect the functioning and quality of life of the affected individuals and their families. Increasing efforts have been made to synthesize evidence and scale up early interventions in community settings [2].

Over the past 2 decades, the field has undergone significant transformations in the delivery of psychological and pharmacological treatments, shifting from face-to-face to incorporating digital technologies as part of mental health care. Such interventions range from the use of web-based platforms and online video conferencing to smartphone apps, the experience sampling method (ESM), and ecological momentary intervention (EMI), as well as chatbots and virtual reality, either as an adjunct to or replacement for in-person care. Each approach may vary in its degree of human contact and intensity [3-7]. Although the application of digital technologies to mental health care has been relatively slow compared to efforts in physical health and behavior change, the emergence of the COVID-19 pandemic has greatly accelerated this process.

### Benefits of Integrating Smartphone-Based Apps Into Mental Health Care

Among the various digital tools, the use of smartphone-based mental health apps (MHapps), specifically blended care, has received increasing scientific attention and support. Some benefits include their ability to save clinicians time, which is crucial given the burden of mental disorders and the persistent challenge of insufficient manpower, as well as to improve treatment accessibility and reduce dropout rates.

### Treatment Adherence and More Sustainable Changes

As in pharmacotherapy, adherence to care plans is a crucial determinant of treatment outcomes in psychotherapy. Using cognitive behavioral therapy (CBT) as an example, adherence includes not only regular therapy attendance but also the completion of homework. Homeworks in CBT are cognitive or behavioral activities (or their combination) that are collaboratively planned between the clinician and service user for completion outside of therapy, which involves the direct application of adaptive strategies into everyday life [8,9]. Such activities are personalized and may include scheduling meaningful activities (eg, “leaving the house for 30 minutes every day”), monitoring daily activities and mood states, and identifying cognitive biases while documenting their relationships with triggering events, mood, and physiological reactions [9,10].

Despite the importance of applying these practices to everyday life for more sustainable treatment outcomes [8], homework nonadherence is profoundly common. Beyond difficulties among the general population [11,12], the maintenance of health-related behavior change can be even more challenging in people with subclinical and clinical mental health symptoms, wherein motivational deficits and anhedonia reflect cardinal symptoms of various psychiatric conditions, such as major depression, schizophrenia spectrum disorders, eating disorders, and posttraumatic stress disorder [13]. Blending MHapps with traditional in-person interventions has the potential to fill this gap.

### The Delivery of In-The-Moment Interventions Beyond the Therapy Room

The portability of smartphones and the lack of geographical limitations in their use have opened new opportunities for more person-centered, holistic, and context-relevant interventions [9,14-16]. The EMI is a novel intervention approach that was built upon the ESM—a self-report diary method that enables the collection of real-time data on experiences within individuals and their interactions with one’s real-world context—by additionally allowing real-world delivery of interventions [17]. The availability of active and passive data collected using smartphone-based EMI provides clinicians with information about service users that is not possible in traditional weekly in-person therapy sessions, such as in-the-moment mood states across everyday life contexts; these data can then be used to identify patterns in mood states and their fluctuations in relation to the context (eg, where the person is, who they are with, and what they are doing) and inform more personalized clinical interventions [17-19].

Of note, although standalone EMIs are associated with reduced psychiatric symptoms compared with those not receiving EMI or other forms of psychotherapy [20], the presence of human contact and support has consistently been observed to yield larger and more robust effect sizes across a range of mental health conditions [3,21], thereby supporting the potential of blended mental health care in improving outcomes.

### Opportunities and Challenges in the Implementation of Blended Care via Smartphone-Based EMI

There is now evidence to support the acceptability and effectiveness of digital mental health interventions in not only high-income but also low- and middle-income countries [22], as well as in those previously thought to be less digitally literate, such as older adults [23]. However, several challenges remain. First, although barriers related to the use of MHapps among users have been explored (eg, limited customizability and technical issues [24,25]), little work has been done to pinpoint the barriers to implementation and its facilitators, particularly with consideration of the wider organizational and societal contexts, as well as the possible strategies to facilitate implementation [26]. The involvement of multiple stakeholders throughout the development and design process of digital interventions can help overcome challenges to their uptake in real-world settings.

Further, most existing MHapps have been either developed for use by specific research or clinical teams [27], for profit, or lack empirical evidence and a theoretical basis [28]. The development of a new MHapp is often not practical, not only in many low- and middle-income countries but also in teams with lower resources and in time-limited settings (eg, context of war, social unrest, and pandemic outbreaks). Free-of-charge and tailorable MHapps or platforms that can be easily adapted are crucial for improving access to personalized health care and contributing to reduced health disparities.

### Objectives

To facilitate advances in the field of digital interventions for mental health, the replicability of both the development and design process is crucial. This paper aims to describe the process of developing a theory-driven, person-centered smartphone-based EMI for use as part of a blended CBT intervention for depression (blended CBT-EMI for depression). Three major phases of the design procedure are described: (1) a review of existing MHapps and “no-code” app development tools for potential adaptation and customization for specific research and clinical purposes, (2) an iterative cocreation process with stakeholders via design thinking workshops, and (3) usability testing. Given that the majority of existing MHapps have been designed for young people, this study involved 2 core groups of older adults as stakeholders in the design process (peer supporters and previous service users), serving as examples for future replications.

## Methods

### Phase 1: Review of Existing MHapps and “No-Code” App Development Tools

Before involving stakeholders in the smartphone-based EMI development process, we conducted an in-depth review of existing digital MHapps to determine whether any platform was readily available for adaptation across clinical populations and settings.

Two reviews of MHapps have been published in the past 10 years (one providing recommendations for future developments of MHapps [16], and another a recent meta-analysis on the efficacy of existing MHapps for depression and anxiety [28]), although neither examined their availability for public use nor their degrees of adaptability. We therefore hand searched all MHapps cited in these 2 reviews on the iOS App Store (Apple Inc) and Android Google Play Store (Google LLC), from which the following information was extracted (as of May 6, 2025): name of the MHapp, availability for download and use, whether it could be adapted by individual researchers or clinicians, key functions, developer, date of last update, and whether clinical trials have been conducted to evaluate its efficacy.

In recent years, the gradual rise of “no-code” app development tools [29,30], which are platforms that allow researchers with no previous software programming experience to build apps via a graphical user interface, has reduced the time and cost required for development. We also reviewed their potential suitability for use compared with readily available MHapps.

### Phase 2: Iterative Co-Design and Cocreation Process of the Platform

After identifying a possible platform for the EMI app, a series of cocreation workshops and usability testing were conducted to ensure the design of the CBT-EMI was person-centered and to minimize implementation barriers in practice.

**Figure 1.**
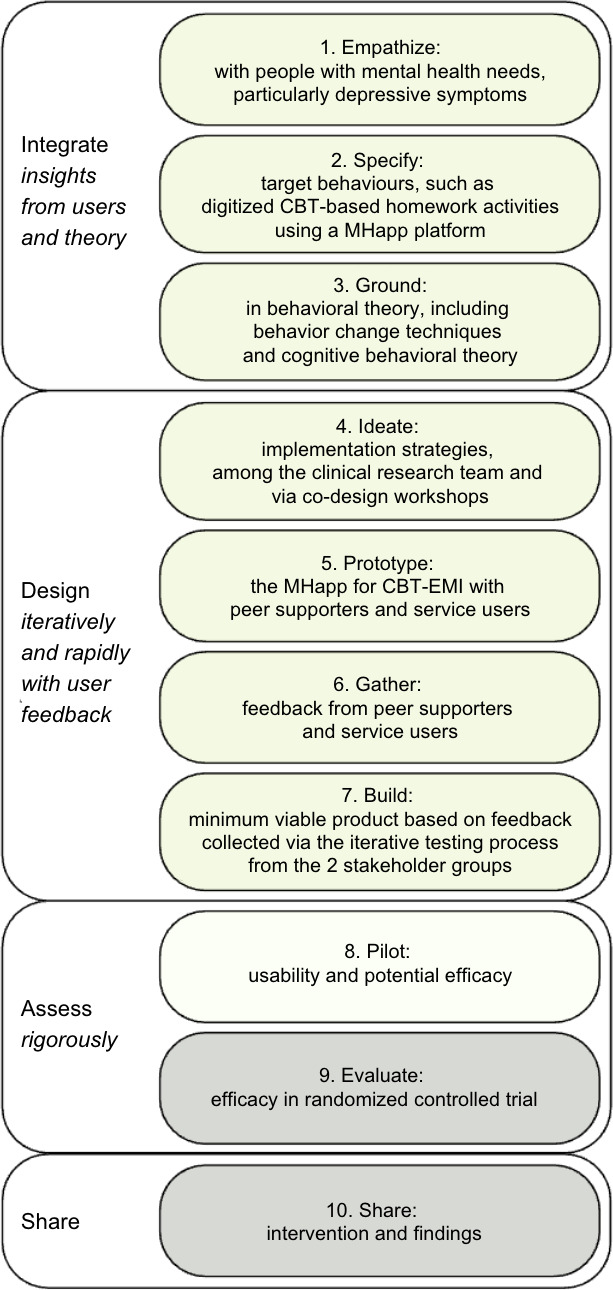
Application of the IDEAS (Integrate, Design, Assess, and Share) framework for developing a blended cognitive behavioral therapy with smartphone-based ecological momentary intervention (CBT-EMI) mobile health app platform.

The IDEAS (Integrate, Design, Assess, and Share) framework [31] was adopted to guide this process. Consolidating strategies from behavioral theory, design thinking, and user-centered design, the IDEAS framework was intended to provide researchers and clinicians with a more systematic, step-by-step approach to developing effective digital interventions that target health behavior change. A total of 10 steps are specified, which can be broadly categorized under 4 stages: Integrate, Design, Assess, and Share ([31] refer to Figure 1).

Similar to a previous study on the development of a mobile app for hypertension prevention [32], this study focuses on the first 2 stages (Integrate and Design) of the IDEAS framework, with preliminary findings evaluated using a minimum viable product for the third stage (Assess). Further evaluation of its feasibility and efficacy will be conducted in the next phase and is beyond the scope of this study.

#### Empathize With Target Users (Step 1)

For this study, target users were older Chinese adults. As such, our smartphone-based CBT-EMI was built upon an existing 6-week, group-based CBT intervention tailored for older adults with subclinical depressive symptoms, which was designed by clinical psychologists and senior social workers as part of a large-scale collaborative stepped-care intervention project, JC JoyAge (Jockey Club Holistic Support Project for Elderly Mental Wellness), across Hong Kong since 2016 [33,34]. The design of the CBT intervention was guided by the ecological validity framework [35,36] to ensure its cultural relevance to the local older adult population, which comprises 8 central elements, namely, language, persons, metaphors, content, concepts, goals, methods, and context. For instance, aside from using traditional Chinese and colloquial Cantonese terms and sentences in all intervention materials and protocols, metaphors (eg, “a broken ship still has three catties of nails,” which is a local idiom meaning “no matter how bad something is, there is still some value in it”) were also commonly referenced to facilitate the relevance of the CBT intervention content to participants. The significance of such cultural adaptations and co-design processes in psychological interventions in the current context has been described in our previous work [33,37-39].

Regarding the EMI platform, we took reference from the existing knowledge base on how digital technologies may be designed to facilitate the older adult population in receiving psychological interventions, particularly in the Asia-Pacific context (eg, the TORCH [Technology provision, On-site technical support, Rehearsal, Connection with group members, and Hardcopy notes] principle [40,41]). To optimize user experience (UX), the platform was also designed according to principles listed in established guidelines on usability testing [42], as well as a guide developed by the local Government of the Hong Kong Special Administrative Region on designing elderly-friendly website or mobile apps (ie, need for clear and easy-to-understand content, easy-to-read-and-operate layout, clear steps and instructions, and support for voice input method [43]).

#### Specify Target Behavior and Ground in Theory (Steps 2 and 3)

Aside from taking reference from the ESM and EMI literature in the design of the CBT-EMI UX, the target behaviors (CBT-based homework) were designed based on (1) theories of CBT, and (2) theories of behavior change and maintenance. We took reference from the traditional printed worksheets used in the regular CBT in JC JoyAge [33,34,44] and pinpointed homework activities for potential application in the blended care.

To optimize more sustainable behavior change (ie, adherence to CBT-based activities between and beyond intervention sessions), we innovatively integrated theories of CBT with the health behavior change literature. Given the increasing emphasis on specifying “active ingredients” to improve the understanding of mechanisms of change in psychological interventions [45], we sought to incorporate behavior change techniques (BCTs) as part of the CBT-EMI, which are known as the smallest replicable components of an intervention for inducing behavior change via specified mechanisms of action [46,47]. The incorporation of such theory-informed BCTs via MHapps may offer an avenue for opening up the black box in mental health science and in advancing person-centered care. Recommendations in the design of EMIs were also reviewed to improve the usability and feasibility of the CBT-EMI platform.

#### Iterative Design Process With Prototype Testing and Feedback Gathering (Steps 4-7)

Building on Steps 1‐3, we recruited 2 groups of core stakeholders to a series of 3 weekly co-design workshops, each lasting 3 hours. The first group was peer supporters aged ≥50 years from the aforementioned project (JC JoyAge [33,48]), who had received >80 hours of training for service provision in the social care setting (October-November 2024). The second group was previous service users of this project who had presented symptoms of subclinical depression (defined as a score of 5‐19 on the Patient Health Questionnaire-9 (PHQ-9) at service intake, February-March 2025). Two mental health researchers (SMYW and MHCI), together with a senior social worker or clinical psychologist experienced in older adults’ mental health care, led the workshops, while another trained research assistant (JKQL) took written field notes of observations and feedback.

All participants were required to have experience in using smartphones and to give informed consent. Those with a history of autism spectrum disorder, intellectual disability, schizophrenia spectrum disorder, bipolar disorder, Parkinson’s disease, or dementia, as well as those with imminent suicidal risk, were excluded. To ensure participants could freely share their ideas and experiences, we opted for around 5‐6 participants per workshop (maximum of 8) with reference to established guidelines on usability testing [42]. Table 1 shows the sociodemographic characteristics of the 2 participant groups.

During Session 1, a series of design thinking activities, such as Empathy Map and the Eisenhower Matrix were implemented to explore the needs of service users, identify pain points, and brainstorm creative solutions (details in Table S1 in Multimedia Appendix 1). Based on the CBT homework and conventional EMI items identified for potential adaptations, the current research team developed a prototype for testing and refinement. Specific considerations were made for the user interface, user design, and interaction flow during the design process (eg, choice of words, use of illustrations, and design style, as well as the number of prompts to be sent per day and the time of sending), which are further discussed and refined throughout Sessions 1‐3 of the workshops.

**Table 1.** Sociodemographic characteristics of participants in the co-design workshops.

Characteristic	Peer supporters (n=8)	Service users (n=5)
Sex, n (%)		
Female	5 (62.5)	5 (100)
Age (years), mean (SD)	61.5 (5.3)	69.8 (3.7)
Highest educational attainment, n (%)		
Primary or below	0 (0)	2 (40)
Secondary school	2 (25)	2 (40)
Diploma	3 (37.5)	0 (0)
University or above	3 (37.5)	1 (20)
Occupation status, n (%)		
Full-time employment	1 (12.5)	0 (0)
Part-time employment or freelance	2 (25)	0 (0)
Retired	5 (62.5)	5 (100)
Health condition, n (%)		
Has any physical health condition	3 (37.5)	4 (80)
Has any diagnosed mental health problems	0 (0)	2 (40)
Severity of depressive symptoms at service intake (PHQ-9)^a^, mean (SD)	—	8.80 (3.03)
Years since peer supporter training, mean (SD)	2.1 (1.1)	—
Smartphone use frequency, n (%)		
1‐2 times per day	1 (12.5)	1 (20)
≥3 times per day	7 (87.5)	4 (80)

a PHQ-9: Patient Health Questionnaire-9.

### Phase 3: Usability Testing of the Blended CBT-EMI Platform

After Session 1, participants were sent a preferred number of prompts per day (as discussed during the workshop) over the subsequent 6 days to test several CBT-based homework assignments in their everyday lives. During Sessions 2 and 3, discussions were then held regarding their experiences with enacting the homework activities over the past week (eg, what they liked about the MHapp design, and what barriers they faced).

The “think-aloud” method was used to obtain participants’ immediate thoughts and feedback on different features of the platform and specifically the following: (1) sampling scheme (including the optimal frequency, length, and schedule of homework activities), (2) preferred wordings and notification settings, and (3) other features considered essential to promote MHapp use (eg, expiration windows and conditional logics). A brief questionnaire was also administered alongside the MHapp demonstration, in which participants rated their preferences for adopting certain features in the final MHapp on a 7-point Likert scale from 1 (strongly disagree) to 7 (strongly agree).

Between Sessions 2 and 3, another round of real-world usability testing of the prototype (refined based on participants’ comments) took place with another set of CBT-EMI homework. During Session 3, final discussions were made to consolidate participants’ preferred features of the MHapp (including logistics), as well as its interface. A final questionnaire, which included 12 statements similar to those in previous work [18], was given to participants at the end of the workshop to capture their thoughts on the functionality of the MHapp and the overall arrangements of the workshops. Each statement was rated on a 7-point Likert scale, from 1 (not at all) to 7 (very).

### Statistical Analysis

All findings gathered from the co-design workshops were synthesized to determine core features to be prioritized in the CBT-EMI platform. Instant data analysis [49]—which is a time-saving approach commonly adopted for usability testing—was performed, wherein 3 researchers (SMYW, MHCI, and JKQL) discussed their observations and field notes after each workshop to identify problems reported by the participants, review action plans, and adjust the prototype of the MHapp for subsequent testing. For any inconsistent suggestions identified from the 2 rounds of workshops held with peer supporters and service users, respectively, the needs raised by the latter group were prioritized. Additional comments were also sought from clinical practitioners and mental health researchers to ensure that the final MHapp design was clinically relevant and feasible in terms of implementation and practice. Descriptive statistics were generated for the quantitative data gathered from the questionnaires on preferences for adopting certain MHapp features, as well as perceived functionality of the overall MHapp and the workshops, using SPSS (version 29.0.1.0; IBM).

### Ethical Considerations

Written informed consent was obtained from all participants. Ethics approval was granted by the Human Research Ethics Committee of the University of Hong Kong (EA240144). Each participant received US $38 (HK $300) after their participation in the 3 co-design workshop sessions to acknowledge their contribution to the co-design process.

## Results

### Phase 1: Landscape Review and Analysis

#### Review of Existing MHapps

A total of 149 unique, named MHapps were identified, among which only 43 (28.9%) could be publicly downloaded. More details of these MHapps, including those that could not be downloaded, are provided in Table S2 in Multimedia Appendix 1.

Most of them were in English, with the exception of 3 MHapps, which were available only in Spanish or Dutch. Of the MHapps retrieved, the majority were available to the public (90.7%, n=39), with access to 4 apps restricted to specific users (ie, US or UK citizens covered by specific employers or health plans, corporations, or study research participants). Since these apps were retrieved from the 2 aforementioned papers, the majority (93%, n=40) had been tested for their efficacy in clinical trials.

Despite the relatively high accessibility to these MHapps by the general public, only 4 (8.3%) could be partially adapted by individual researchers or clinicians (eg, notification settings and therapeutic activities for clients could be customized, and data analytics could be retrieved from the backend server). Notably, only existing content made available by the developers could be used and could not be edited, meaning that no new content relevant to specific interventions or contexts could be incorporated.

#### Review of Existing “No-Code” App Builders

Since none of the reviewed MHapps could be fully tailored for use in our local context, we reviewed the potential of adopting “no-code” app builders for designing the blended intervention. A recently published review has identified 11 such tools [29]. Among them, only 1 was designed primarily for mental health research and interventions (m-Path, initially developed by the KU Leuven team in Belgium), which appears to be suited for our intervention purposes given the following: (1) features available for ESM and EMI (eg, a wide range of mental health–related question types available, allows collection of both active and passive data, and includes gamification options); (2) core aspects of its interface could be personalized by individual users (eg, language, color, and text); (3) no coding experience required (eg, web-based interface with a summary dashboard of all data points and events created by each user for review by practitioners or researchers); (4) tailorable in multiple aspects, including survey content (eg, multiple question and response types available), survey flow (eg, randomization and conditional logic options), and study design (eg, event-contingent or time-based sampling, personalized reminders); (5) strict data security and privacy policies (eg, no mandatory disclosure of personal particulars during app registration, anonymous data processing and storage, end-to-end phone-server communication, and compliance with standardized regulations, allowing participants to delete or block researchers’ access to their data at any time); and (6) compatible with both iOS and Android devices [50].

A further review of the literature suggested that previous mental health studies have chosen m-Path given its low barrier to use (eg, no personal data such as email, phone number, or name is required [51]). We also confirmed that existing no-code app builders, except m-Path (at least up to the time of paper preparation), were primarily targeted at changing and tracking physical activities (eg, step trackers) and did not include the above features. We therefore selected m-Path as the platform to build the present blended CBT-EMI MHapp for delivering CBT-based homework using EMI to manage and reduce depressive symptoms. The procedures adopted in this study in co-designing the blended care platform, nevertheless, should provide a helpful reference for future work if another platform is identified as more suitable.

### Phase 2: Iterative Co-Design and Cocreation Process of the Platform

#### Empathize With Target Users

Although m-Path was already available in 16 languages at the time of exploration, the platform was not available in Chinese. While the majority of residents in Hong Kong are proficient in English, the prevailing spoken language remains Chinese, particularly among older adults. We therefore reached out to the m-Path team to discuss the possibility of translating the platform into traditional Chinese (participants could still freely choose to use the platform in English or other languages, although the traditional Chinese version may provide more personal relevance, particularly for intervention purposes). Two members of the current research team (SMYW and JKQL), who have experience with translations in previous work and are proficient in Chinese and English—both of which are official languages in Hong Kong—independently completed the translation process from the landing pages to all subsequent pages. As the CBT program had already been developed following cultural adaptation frameworks in JC JoyAge, its CBT-based homework activities were directly incorporated into m-Path, with only minor adaptations made to facilitate reading on smartphones. Clinical psychologists of the project subsequently reviewed all materials to ensure the core therapeutic components were retained in the homework activities.

To ensure the accuracy and relevance of the translated materials, participants were presented with the initial research-adapted version of CBT-EMI and were then invited to reflect on and discuss the understandability and cultural friendliness of the terms and the illustrations. Adaptations were made iteratively and concluded when participants were satisfied. During this process, participants generally expressed satisfaction with the overall flow of CBT-EMI. Several issues related to language and context within the cultural adaptation framework were identified and addressed. Regarding language, the entire intervention was revised from written Chinese to spoken Chinese. Specific terms for emotional expressions were replaced with colloquial expressions, with additional examples relevant to older adults incorporated. For instance, scenarios such as “having conflicts with family members, neighbors, and friends” were added to the “interpersonal relationships” category in the Mood Check exercise.

#### Specify Target Behavior and Ground in Theory

##### CBT Theory

With reference to the design of the pre-existing CBT for depression in older adults in Hong Kong [33,34], a series of possible homework activities grounded in CBT theory were identified for incorporation within the blended care, as follows: Mood Check, Relaxation Exercises, and Behavioral Activation (engage in relaxation exercises and participate in enjoyable, mood-inducing activities in daily life), “Hot Cross Bun” diagram (map triggering event, thoughts, feelings, physical sensations, and behaviors), Gratitude Diary (identify 3 things or people that one is grateful for on the day), Activity Scheduling (preplan mood-inducing activities that one can engage in during the week and record mood afterwards), and Wellness Toolbox (preset actions to engage in when feeling distressed in the future).

We strived to resemble the homework activities from the original paper-and-pen version by preserving the question type, multiple-choice options, and their overall length. Clinical psychologists reviewed the content of the homework to ensure the therapeutic quality of the app-adapted homework.

##### Health Behavior Change Theory

Further, we drew on the Health Action Process Approach (HAPA [52]) in modifying the intervention, both in terms of how the CBT homework activities can be delivered via m-Path, as well as the content of the in-person sessions. This was considered relevant, particularly given the challenges often observed in treatment and homework adherence in regular CBT.

The HAPA is one of the leading models informing the processes underlying health behavior change and maintenance. Accordingly, the combination of goal setting, planning, and perceived self-efficacy plays a crucial role in determining whether one will initiate a behavior for health changes and maintain its enactment in the long term [52]. Three phase-specific forms of self-efficacy are specified: action self-efficacy, which occurs during the motivation phase when a person has not yet enacted the behavior but develops an intention, even if it requires planning and a lifestyle change, as well as maintenance and recovery self-efficacy, which occurs during the volitional phase when the behavior has already been enacted but determines whether the person can continue to adhere to the behavior even if there are hurdles and can get back on track even after having derailed, respectively [53].

With reference to the health behavior change literature, we therefore identified several BCTs targeting “beliefs about capabilities” (a mechanism of action conceptually identical to self-efficacy [54,55]) in the design of the CBT-EMI platform. The underpinning assumption is that participants will show greater motivation in initiating and continuing the enactment of CBT-based homework activities in their everyday life via incorporating BCTs that foster self-efficacy using the EMI method, which, in turn, results in greater homework adherence, greater improvements in depressive symptoms, and a lower rate of relapse. Table 2 provides an overview of the mapping of BCTs and CBT-based homework in the proposed blended care intervention design. The BCT clusters, mechanism of action, and definitions of BCTs are based on the works by Michie et al [46] and Carey et al [54].

**Table 2.** Selection of self-efficacy–related behavior change techniques and their application in the blended CBT-EMI.

BCT^a^^b^ (cluster)	Definition of the BCT	Application in the blended CBT-EMI^c^ (implemented in a CBT session or EMI)
Set graded tasks (repetition and substitution)	Set tasks with the client, from simple to increasingly difficult (but achievable), until target behaviors are performed.	The intensity and frequency of homework increased throughout the 6-week CBT intervention. For example, after Session 1, clients were asked to complete “Mood Checks” via the smartphone MHapp twice a day before Session 2. After Session 2, clients were tasked with “Mood Checks,” followed by in-the-moment behavioral activation, and so on. New homework was added or updated in the following weeks (except for “Mood Check,” which was prompted throughout the 6 weeks) [EMI].
Verbal persuasion about capability (self-belief)	Encourage the client to trust their capabilities in performing the behaviors and in overcoming self-doubt.	Across the various CBT sessions, the practitioner discussed any concerns the clients had and assisted them in developing more confidence in their capabilities in making changes in the behaviors that are anticipated to have benefits for their mental well-being and performing them. Examples of verbal persuasion include the provision of validation to participants’ experiences, including feelings, thoughts, and behaviors, reframing and challenging negative thoughts, as well as evaluating behaviors and progress in subsequent sessions [CBT session].
Focus on past success (self-belief)	Facilitate reflection on and list previous successes in performing the exact or similar behaviors.	Aside from discussing problems and identifying risk factors, the practitioner facilitated each client in becoming aware of their personal strengths and protective factors, including examples of their previous experiences in enacting the CBT-based homework and in overcoming challenges in performing them [CBT session].
Problem solving (goals and planning)	Facilitate the client in analyzing factors that might influence the (non)enactment of behaviors and develop strategies to overcome barriers and increase facilitators (eg, coping planning and relapse prevention).	The practitioner discussed the core “Hot-Cross Bun” model of CBT, which highlights the intricate relationships between an external trigger, as well as thoughts, feelings, physical or physiological sensations, and behaviors; applied it to the formulation of each client’s experiences, and considered the 5Ps of their experiences (presenting problems, predisposing factors, precipitating factors, perpetuating factors, and protective factors). These clinical formulations then formed the basis for clients to identify the personal meanings behind the enactment of homework activities, as well as strategies to reduce barriers and increase chances of successful enactment [CBT session].
Demonstration of the behavior (comparison of behavior)	Provide an example of how the behaviors can be performed, either directly or indirectly (eg, via photos or videos), for the client to learn.	The practitioner demonstrated all homework activities during the CBT sessions, including a run-through of each procedure. Each activity was completed by the clients at least once before leaving the therapy room. All activities on the smartphone MHapp were designed to be straightforward. For more complex tasks, guided examples were incorporated into the MHapp (eg, video-guided diaphragmatic breathing exercises) [CBT session and EMI].
Instructions on how to perform a behavior (shaping knowledge^b^)	Provide details on how each specified behavior is performed.	The practitioner provided details on how each homework activity on the smartphone MHapp was provided during the CBT session (via demonstration, as above, and with handouts as needed). Additional descriptions were also provided in the MHapp for more complex homework activities (eg, Wellness Toolbox) [CBT session and EMI].
Behavioral practice or rehearsal (repetition and substitution)	Prompt the practice or rehearsal of the behavior at least once in a context or time when its performance is not necessary to develop it into a habit.	Using the EMI, clients were prompted to complete CBT-based homework activities via the smartphone MHapp (instead of the traditional paper form) at least twice a day, once in the morning and once in the evening, in their everyday lives to ensure their implementation across various settings and mood states, including during times of relatively low negative affect, to develop it into a habit. The EMI prompt was also delivered and rehearsed before the end of each CBT session to facilitate clients in familiarizing themselves with the process [CBT session and EMI].
Reduce negative emotions (regulation)	Provide advice on methods to reduce negative emotions to facilitate the performance of behaviors.	The practitioner introduced possible strategies to reduce negative emotions (including sad mood) during the CBT sessions, with the EMI designed to prompt behaviors that facilitate in-the-moment reflections of one’s mood states across social contexts and improve mood [CBT session and EMI].

a BCT: behaviour change technique.

b All BCTs listed are postulated to influence behaviors via the MoA “Beliefs about capabilities,” except “Instructions on how to perform a behavior” (with mechanism of actions “Knowledge” and “Skills”), which was also coded as recommended since instructions were provided alongside the BCT “Demonstration of the behavior.”

c CBT-EMI: cognitive behavioral therapy with smartphone-based ecological momentary intervention.

### Iterative Design Process With Prototype Testing and Feedback Gathering

**Figure 2.**
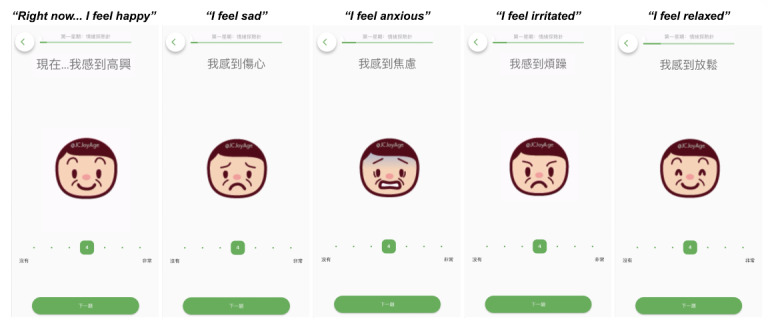
Example of the CBT-EMI (cognitive behavioral therapy with smartphone-based ecological momentary intervention) MHapp prototype: Mood Check (illustrations by an experienced illustrator, Ms. Coco Lee, as part of the intervention service project).

A prototype of the CBT-EMI, incorporating the various CBT-based homework activities, was developed using the m-Path platform (refer to Figures2^3^). The overall flow and user-friendliness of the platform were first iteratively tested within the research team before discussions with the 2 stakeholder groups.

**Figure 3.**
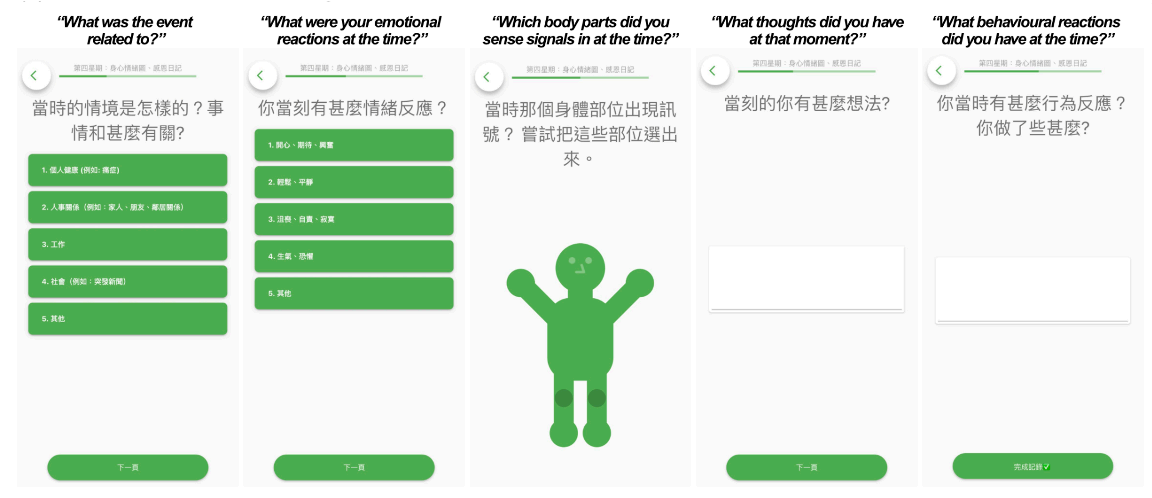
Example of the CBT-EMI (cognitive behavioral therapy with smartphone-based ecological momentary intervention) MHapp prototype: Hot Cross Bun.

To ensure all participants sufficiently understood how the CBT-EMI platform operated, all homework activities were tested in a separate group of 5 older adults before the process of iterative testing by participants between sessions, with detailed explanation of different features of m-Path in Session 2 (in line with other components of the TORCH principle, namely “on-site technical support,” “rehearsal,” and “connection with group members” [40]). Hardcopy notes were not provided in this series of workshops since all details were discussed together in the small-group setting, although they are intended to be incorporated into the implementation of CBT-EMI. To ensure equity in access to the CBT-EMI and the co-design process, smartphones were available for those whose devices were not functioning (ie, “technology provision” component of the TORCH principle [40]), although all participants used their personal smartphones throughout the 3 workshop sessions, suggesting their ubiquity even in the older adult population today.

### Needs and Priorities in the Design for Blended Care

**Figure 4.**
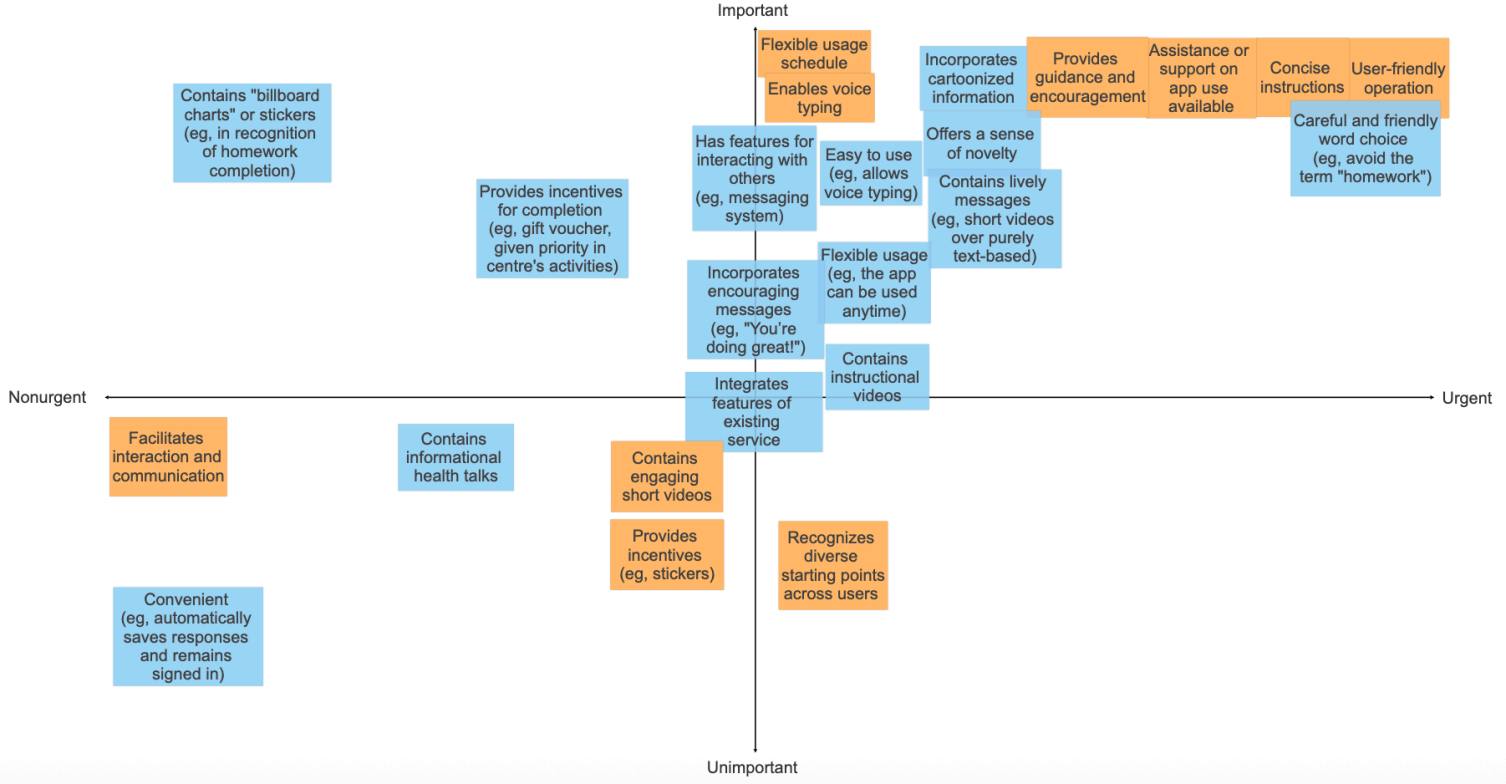
Prioritization of mental health app features using the Eisenhower Matrix by peer supporters (in blue) and service users (in orange).

Following the Empathy Map activity, the set of needs identified by both peer supporters and service users was respectively mapped and positioned on an Eisenhower Matrix to determine their relative urgency and importance for adoption (Figure 4). The mapping of these needs on this 2×2 matrix enabled a clear visualization of how some needs were considered urgent or important by both stakeholder groups, while some were considered only urgent or important by peer supporters and not service users, and vice versa. Examples of how the MHapp could integrate features of existing services include the use of the PHQ-9 for screening and assessing depressive symptoms, as well as the use of the Wellness Recovery Action Plan to facilitate service users in developing personalized wellness plans. Users had diverse starting points, including differences in digital literacy, mental health literacy, and mental health needs.

All needs positioned in the “urgent-and-important” quadrant were adopted and implemented in the MHapp in this study. The only exceptions were those that were raised specifically by peer supporters but considered neither urgent nor important by service users.

For instance, ensuring a “user-friendly operation of the MHapp,” together with the “use of careful and friendly word choices,” was considered both urgent and important by both stakeholder groups and were principles we followed in designing the EMI platform. This was supplemented by the suggestion of providing concise instructions, offering assistance and support on the MHapp, alongside providing guidance and encouragement to participants from service users’ perspectives. Nevertheless, whereas peer supporters considered the term “homework” to be discouraging, this was not seen as a problem by service users. Similarly, incorporating more lively messages (such as adding short videos) and features that allow interactions among users to encourage MHapp use were considered helpful by peer supporters but not service users. Since these were also features that demanded relatively more technical support, we considered these as “good-to-have” features but that could be given a lower priority at the current stage of MHapp development.

**Figure 5.**
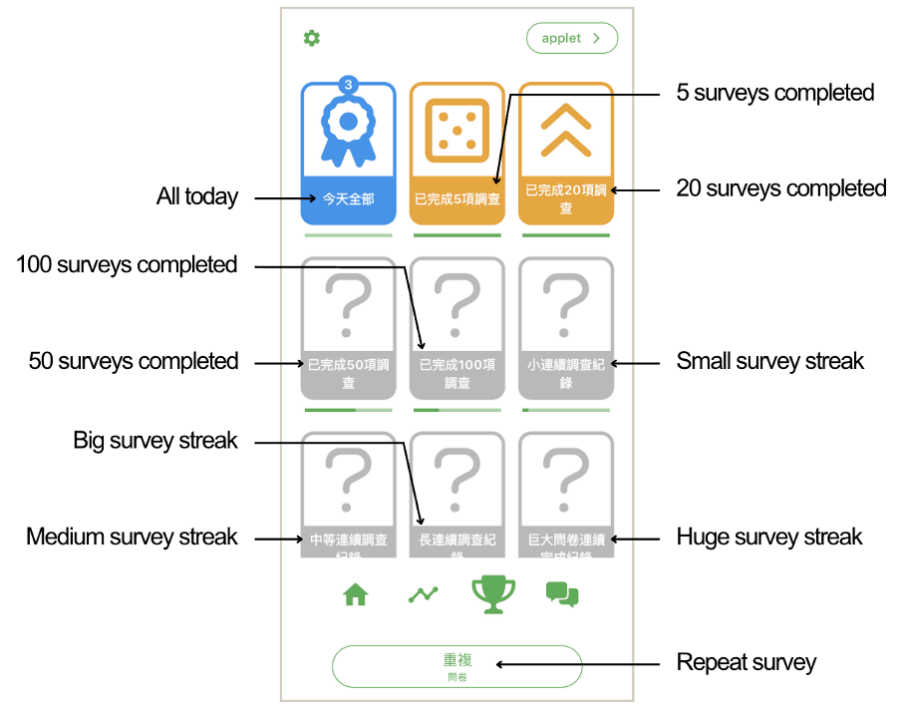
Example of the “award page” in the CBT-EMI (cognitive behavioral therapy with smartphone-based ecological momentary intervention) MHapp.

For needs identified to be urgent-and-unimportant and not–urgent-and-important, we considered their feasibility and discussed strategies to make their implementation possible. As an example, “recognizing different starting points across users” (considered urgent by service users) could be addressed by ensuring sufficient guidance and support are given to participants during the CBT-EMI, both in terms of technical and mental health support, together with an emphasis on person-centered care throughout the intervention. Further, while developing a “billboard chart” to motivate homework completion may not be fully feasible at the current stage (considered important by peer supporters), one means of achieving this aim was through providing virtual “awards” as an incentive. With the “award” function on m-Path, participants can receive virtual badges after reaching preset goals (eg, completing all beeps in a day, completing 10 surveys in total, and so on). Users can view all awards they have accumulated on their personal “award page” (refer to Figure 5).

“✓” represents that the MHapp has been adapted based on specific user feedback and needs, “x” represents that no adaptation was made after reconsideration of the feedback, “n/a” represents that adaptations could not be made by individual researchers or clinicians (eg, changes only possible by the developers, namely the m-Path team in this context), and “/” represents that the feedback could not yet be incorporated at the current stage due to manpower and resource considerations but may be considered at a later stage and by other research teams.

### Adaptations Made to the CBT-EMI MHapp

Table 3 presents the needs and feedback generated by peer supporters and service users during Sessions 2 and 3 of the co-design workshops and whether adaptations were made accordingly. Details of how the CBT-EMI was adapted are provided in Table S3 in Multimedia Appendix 1. A total of 40 needs were summarized and categorized into the following: sampling scheme (n=6), question types (n=6), app design and UX (n=14), homework-specific feedback (n=8), and other feedback beyond the app (eg, “Incorporate additional human support” [n=6]).

**Figure 6.**
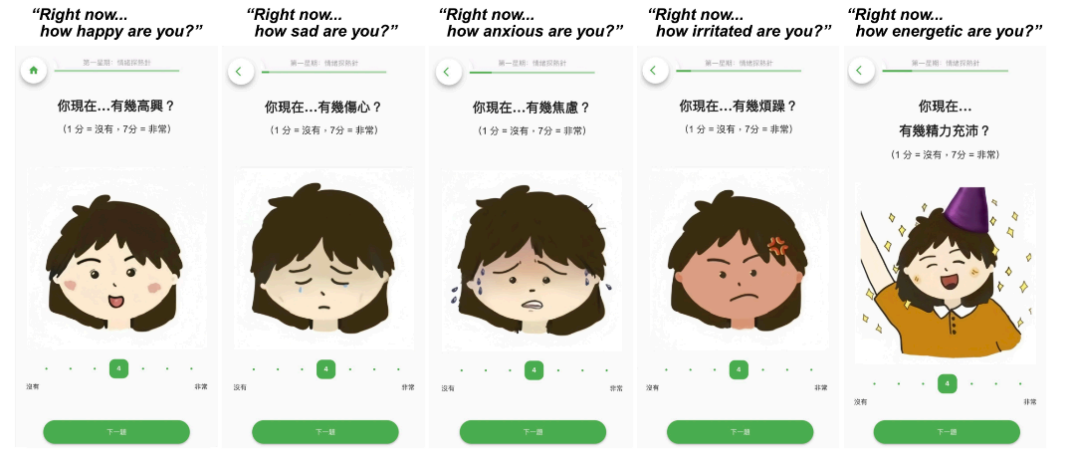
Example of the refined version of the CBT-EMI (cognitive behavioral therapy with smartphone-based ecological momentary intervention) MHapp: Mood Check (illustrations by the first author, Stephanie MY Wong).

**Figure 7.**
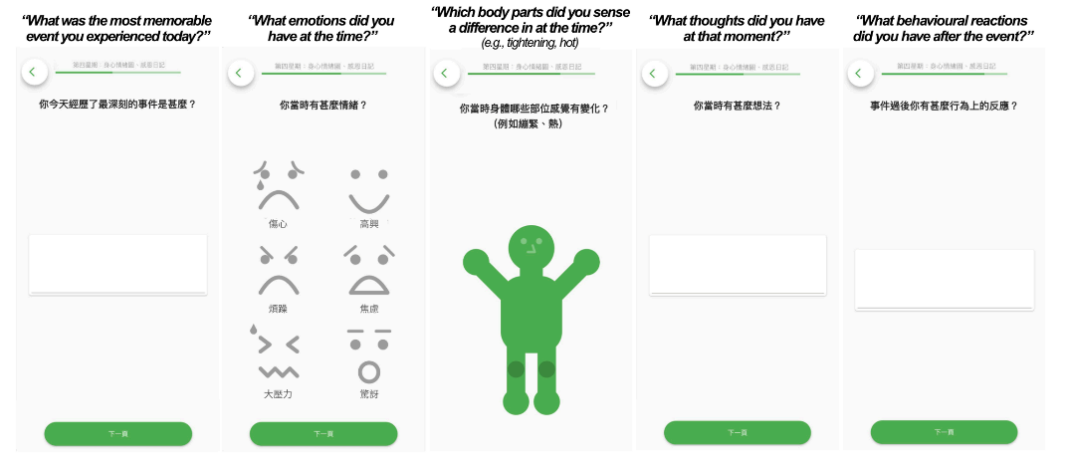
Example of the refined version of the CBT-EMI (cognitive behavioral therapy with smartphone-based ecological momentary intervention) MHapp: Hot Cross Bun.

Of these, 24 were from peer supporters, 7 were from service users, and 9 were from both stakeholder groups. The majority of these needs could be directly adapted for incorporation into the CBT-EMI (62.5%, n=25), and 17.5% (n=7) could not be directly adapted by individual researchers or clinicians on the platform (ie, requires amendments by the developer). A small proportion (10%, n=4) were not adapted, predominantly due to clinical and theoretical reasons (eg, since the intention of applying EMI to CBT was to encourage the enactment of CBT-based homework across settings and mood states in the real world, the prompting of 2 EMI beeps per day was retained rather than adjusting the goal to completing 12 beeps in total before the next session). For all feedback that was not adopted, we ensured the feature was acceptable to service users in the second round of workshops. An additional 4 needs identified were considered important for future blended care developments but have yet to be incorporated into the present CBT-EMI at this stage due to manpower and resource considerations (Table 3). Based on these needs and feedback, the MHapp was refined, with examples shown in Figures6^8^ (eg, “adopt briefer question types,” “make the questions and prompts more straightforward,” and “use figures and icons that are more age-inclusive and appear less old). For instance, in the Mood Check exercise (Figure 6), illustrations were replaced with more age-inclusive and youthful designs based on participants’ feedback, with additional details provided to denote what the lowest and highest values on the slider represent. In the Hot Cross Bun exercise (Figure 7), the triggering event item was amended from a multiple-choice to an open-text response, while the emotions item was replaced by the multismiley question. The amendments made to the interface were also in line with previous UX design guidelines [42,43].

**Table 3.** User needs and feedback, and adaptations made.

User needs and feedback		Source	Adapted?
Sampling Scheme			
1.	Enable customization of the number of beeps for EMI^a^ reminders to enhance flexibility.	PS^b^	Yes
2.	Schedule 2 beeps a day (1 before noon and 1 at night).	Both	Yes
3.	Enable flexibility in responding to EMI beeps, allowing participants to complete the questionnaire later after the beep time.	Both	Yes
4.	Require the completion of a set number of EMI activities (12 in total, without specification of time or day of completion) in the subsequent 6 days before the next CBT^c^ session, rather than completing 2 EMI beeps per day.	PS	No
5.	Enable participants to mute the beeps if needed.	PS	No
6.	Implement only Mood Check across all beeps to shorten overall EMI completion time.	PS	Yes
Question Types			
7.	Insufficient options in the multiple choice questions (eg, relaxation exercises and activity logging), and add an ”Other” and a free-text response option for participants who want to document their experiences in greater detail.	PS	Yes
8.	Rearrange the question flow such that questions likely to prime negative emotions are placed at the beginning.	PS	Yes
9.	Provide suggested statements for open-text questions as prompts for responses to reduce the level of difficulty.	PS	Not applicable
10.	Adopt the photo-upload question type (”Plus Point”), which appeared interesting, although the term should be amended as it is slightly confusing in this context.	PS	Yes
11.	Adopt briefer question types (eg, multiple choice questions).	SU	Yes
12.	Allow users to select multiple options in multiple choice questions to enhance their functionality.	SU	Yes
App Design and User Experience			
13.	Make the questions and prompts more straightforward (eg, questions to be more specific rather than generic).	SU	Yes
14.	Use figures and icons that are more age-inclusive and appear “less old.”	PS	Yes
15.	Develop 2 interfaces (1 for young-old users and another for old-old users).	PS	Not applicable
16.	Incorporate a daily check-in element with encouraging messages (eg, on the home screen) to motivate participants to develop a habit of completing MHapp exercises.	PS	Not applicable
17.	Customize reminder messages to make them more engaging and attention-grabbing so that participants will notice and respond.	PS	Yes
18.	Enable users to refine their answers to previous questions in the same beep.	Both	Yes
19.	Incorporate a clearly labeled “back” button for users to move through the MHapp.	PS	Not applicable
20.	Increase button sizes within the app to accommodate smartphone screens with lower sensitivity, making it easier for users.	PS	Not applicable
21.	Add a magnifying function for participants when clicking on an image.	PS	Not applicable
22.	Implement more customized profile settings by allowing users to upload profile pictures.	PS	Not applicable
23.	Add a button popping up at the front page that activates upon completion of exercises (eg, Daily Challenge) and notify participants when exercises are completed or pending.	PS	Not applicable
24.	Visualize upcoming scheduled exercises to keep users engaged and motivated, preferably in a calendar view.	PS	Not applicable
25.	Incorporate a “prize” function with a “congratulatory sound” upon completion of an exercise.	PS	Yes
26.	Highlight that the information is from credible sources when presenting materials and exercises to address concerns about fake news and scams online.	Both	Yes
Homework-specific: Mood Check			
27.	Figures that indicate emotions should be more explicit, such as using more obvious signs for an angry face.	PS	Yes
28.	Provide more explanation on what each of the emotions means (eg, what is meant by “anxiety”), as simply selecting a score cannot help the user reflect on why those emotions occur.	PS	Yes
29.	Even though labels are provided below the anchors (1 and 7), it was unclear what the low and high scores refer to, making it difficult to accurately reflect one’s mood states.	SU	Yes
30.	Asking about mood states within a 1-hour timeframe may be too short, as events that occurred earlier could still impact a person’s mood states throughout the day or even across multiple days.	SU	Yes
Behavioral Activation			
31.	Incorporate additional multimedia elements, such as short videos (eg, guided mindfulness videos) with regular updates.	Both	Yes
32.	Incorporate a wide range of easy-to-digest reading materials within the MHapp, allowing users to read at their own convenience.	Both	Yes
”Hot Cross Bun” and Gratitude Diary			
33.	Provide more space to describe the triggering events (lack of space for writing in the MHapp prototype).	SU	Yes
34.	Requiring the documentation of 1 thing or person to be grateful for instead of 3 would be more manageable. Briefly recording it on some days across a week, rather than demanding a detailed account on a specific day, will be preferable.	SU	Yes
Feedback beyond the MHapp			
35.	Instead of beeping, users using the built-in reminder from the system, SMS text messages, or WhatsApp reminders could be incorporated to increase the completion rate.	Both	Yes
36.	Provide a contact point for initial demonstration of the app and ongoing troubleshooting.	Both	Yes
37.	Incorporate additional human support (eg, from peer supporters) to guide users in using the app and check-ups for those who did not respond (eg, after 2 full days).	Both	Not applicable
38.	Match accounts to family members or peer supporters so that they can check users’ records regularly.	PS	Not applicable
39.	Make the app more multifunctional (instead of a stand-alone app just for mental health), including a function to connect with others.	PS	Not applicable
40.	Offer tangible incentives beyond the MHapp, such as financial incentives, to increase their motivation.	PS	Not applicable

a EMI: ecological momentary intervention.

b PS: peer supporter.

c CBT: cognitive behavioral therapy.

**Figure 8.**
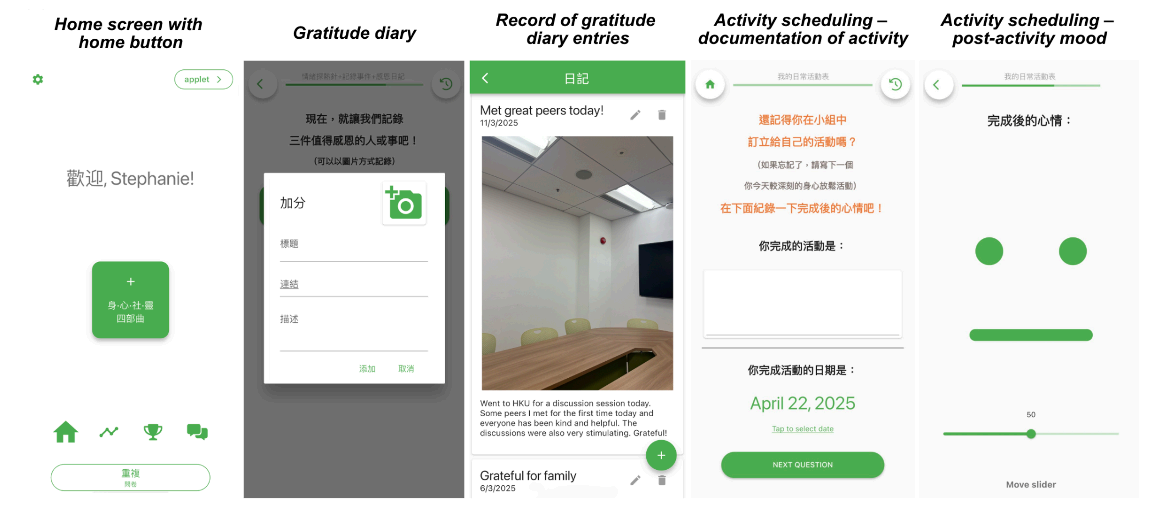
Example of the refined version of the CBT-EMI (cognitive behavioral therapy with smartphone-based ecological momentary intervention) MHapp: Other core activities, such as the Gratitude Diary and Activity Scheduling.

### Usability Testing and User Preferences

Based on the ratings given by both peer supporters and service users, the enabling of multiple answers was generally preferred in multiple choice questions over a single answer (Table S4 in Multimedia Appendix 1). Other features of the MHapp platform were generally well accepted by both peer supporters and service users, although service users generally did not show a preference for the use of the multismiley question and instead preferred separating the various mood states into separate questions in the Mood Check. This question type was therefore only adopted for the CBT-based Hot Cross Bun activity, which was recommended by service users during the workshop.

In addition to adapting specific question types, both peer supporters and service users emphasized the importance of initial demonstration and ongoing technology support. As older adults may encounter challenges when learning new technologies, we made additional effort during app deployment to facilitate this process (eg, providing a step-by-step guide for downloading and setting up accounts, a brief walk-through of MHapp’s built-in security policies, and an ongoing contact point for troubleshooting and support).

In terms of the overall acceptability and feasibility of the MHapp for CBT-EMI, both peer supporters and service users found the m-Path platform to be easy to control and use, and both the texts and instructions were clear (Table 4). Possibly because service users had previous experience in engaging in CBT or other intervention groups, their ratings were slightly lower than those of peer supporters on items related to whether the EMI questions were difficult or unclear (mean 1.40, SD 0.55 vs mean 2.88, SD 1.55), disturbance related to the number of beeps per day (mean 1.20, SD 0.45 vs mean 2.38, SD 1.51), as well as the time needed to answer the questions per beep (mean 1.40, SD 0.55 vs mean 2.88, SD 1.64). Overall, both groups of stakeholders were supportive of the integration of the m-Path platform into routine therapeutic groups to improve the application of skills learned in everyday life and also reported enjoying the co-design workshops (Table 4).

**Table 4.** Acceptability and feasibility of the blended cognitive behavioral therapy with smartphone-based ecological momentary intervention (CBT-EMI).

Acceptability and feasibility (1=not at all, 7=very)	Peer supporters (n=8)	Service users (n=5)
App-related, mean (SD)		
Was the m-Path difficult to control? (reversed)	1.38 (0.52)	1.20 (0.45)
Was it difficult to switch on the m-Path app? (reversed)	1.38 (0.52)	1.00 (0.00)
I found the m-Path app easy to use.	6.00 (0.93)	6.00 (0.71)
EMI content and design-related, mean (SD)		
Was the text on the screen readable and clear?	6.00 (0.76)	6.40 (0.55)
Were the verbal instructions you received about using m-Path clear?	5.38 (2.00)	6.20 (0.45)
Were the written instructions you received on m-Path clear?	5.88 (1.46)	6.20 (0.84)
Were the questions you answered on m-Path difficult or unclear? (reversed scored)	2.88 (1.55)	1.40 (0.55)
Did you find it annoying or stressful to use m-Path with respect to..., mean (SD)		
…the number of beeps per day? (reversed scored)	2.38 (1.51)	1.20 (0.45)
…the time it took to answer the questions for a single beep? (reversed scored)	2.88 (1.64)	1.40 (0.55)
…the noise or sound volume? (reversed scored)	1.75 (1.16)	1.20 (0.45)
Blended care–related, mean (SD)		
I agree to integrate m-Path into routine therapy groups to provide an opportunity to apply the skills learned in the groups to daily life.	6.25 (0.71)	5.60 (1.14)
Overall workshops, mean (SD)		
I enjoyed the process of the 3 co-design workshops.	6.38 (0.74)	6.60 (0.55)

## Discussion

### Principal Findings

The integration of digital-assisted support, such as smartphone apps, into traditional in-person psychological interventions is a promising direction toward improving mental health outcomes. Yet, the challenges posed by the development of new MHapps for specific populations, including not only the high costs involved but also the need for specialized manpower and time, make the implementation and scaling up of blended care with smartphone-based technologies difficult. A potential option is to use existing MHapps; yet, our review showed that none of the 149 possible apps supported the full content adaptation necessary for localized, culturally responsive interventions. This observation highlights a significant gap in the market and underscores the need for highly adaptable and customizable platforms.

Leveraging existing no-code app development platforms, our study demonstrated the feasibility of integrating smartphone-based EMI with traditional CBT via a phased, methodologically rigorous approach. Specifically, the final platform incorporated core CBT-based activities, such as “Mood Check” and “Gratitude Diary,” which are aligned with established mechanisms of change (eg, behavioral activation and activity scheduling [44]) and the HAPA [52,53]. Evidence-based BCTs targeting self-efficacy were also incorporated to improve the enactment of CBT-based homework and its sustainment in everyday life contexts. By describing this iterative design and development process in detail, such culturally adapted, person-centered blended mental health care models have the potential for implementation not only for older adults in Hong Kong but for broader populations and contexts, including time- and resource-limited settings.

Using co-design activities, such as the Eisenhower Matrix to prioritize feedback, 40 user needs were identified—62.5% of which were implemented. These included simplified instructions and interface, the use of friendly language, and gamified elements (eg, virtual badges to reinforce engagement), which are also key features identified as important by users in other studies with young people [56,57] and are incorporated as part of the recommendations for MHapps in a recent review [16]. While some suggestions were deferred due to technical or clinical constraints, stakeholder evaluations rated the platform as intuitive and clinically relevant. Preferences such as separating mood-state questions (rather than combining them into a single scale) were particularly noted. Both peer supporters and service users rated the platform highly for ease of use and clarity. Importantly, the platform demonstrated strong feasibility for integration into group therapy settings to reinforce real-world application of therapeutic skills. Overall, blended CBT-EMI interventions appear promising for therapeutic engagement, homework adherence, and symptom management, particularly among older adults in our study context.

### Strengths and Limitations

This study has important implications for researchers, clinicians, and digital health developers. A major strength lies in its systematic and theory-driven development process. The phased methodology—comprising needs assessment, platform selection, cultural and linguistic adaptation, and co-design—offers a replicable model for future interventions. Theoretical integration is another notable strength; the intervention is grounded in CBT and enriched by HAPA, aligning clinical techniques with behavioral science to support sustained change. The detailed mapping of how various BCTs were incorporated in the CBT-EMI enables a more fine-grained investigation of their independent effects on clinical outcomes in future studies, which can be conducive to efforts in the identification of “active ingredients” in psychological interventions [45].

The blended care intervention we presented further benefits from its strong cultural relevance. Stakeholder engagement was central to the adaptation process, ensuring the platform was sensitive to local needs and user expectations. By addressing user interface design, motivational features, and language preferences, we were able to develop a culturally attuned and user-friendly solution for mental health care in the real-world setting. These features are critical for maintaining long-term engagement among users. The co-design process also greatly facilitated stakeholder-driven adaptations and potentially enhanced the feasibility of implementation in routine care.

Nonetheless, we note several limitations. While the Mood Check items we adopted are similar to those commonly used in ESM and EMI among adolescents and adults, the present blended care platform was co-designed only with Cantonese-speaking older adults in Hong Kong. Additional rounds of co-design processes may be helpful to ensure its potential application to other age, cultural, or linguistic groups. In particular, we highlight the significance of considering the use of culturally sensitive mediums [58], local languages and dialects [59], as well as barrier-free design (eg, allowing audio input as an alternative to text-based options [60]). While the sample size of this study is in line with guidelines on performance-based usability testing [42] and is comparable to other studies using co-design methods in the development of health interventions [61,62], we acknowledge that perspectives beyond the 13 participants involved may not be captured (eg, those with less motivation to participate in the study). We did, nevertheless, aim to include participants with more diverse sociodemographic backgrounds and experiences in psychotherapy to capture a wider range of perspectives. Whether additional amendments may be needed to improve the usability of the platform for a majority of the population would require further exploration.

We also note that several user-identified needs were not implemented due to platform limitations, pointing to future opportunities for enhanced collaboration between researchers and technology developers. Given the lack of no-code app platforms available that were specifically designed for EMI, we selected m-Path for building the EMI for the present blended care. We acknowledge that the focus on a single vendor could have possible issues related to the long-term sustainability of the CBT-EMI. Yet, the aim of this study was to provide a transferable framework that could guide researchers or stakeholders in the co-design of a digital mental health platform, particularly in settings where the development of a new app is not feasible. The insights we gathered into the prioritization of MHapp features and user needs also offer a reference for future studies in the design of more age-friendly MHapps targeting the older adult population. Given the relatively short study period, we cannot determine whether individuals would continue using the platform beyond the intervention period. Whether individuals could successfully develop habits in applying CBT-based techniques in their everyday lives without the continued use of the platform postintervention would be an area for future investigation.

In line with the IDEAS framework [31], empirical validation through randomized controlled trials with a longitudinal follow-up would be an important next step to confirm the efficacy of the CBT-EMI in terms of symptom reduction, adherence, and long-term outcomes. In particular, it would be worthwhile to examine whether people receiving the novel blended care—as informed by both cognitive-behavioral and health behavior change theories—will show greater symptom improvements and higher rates of clinical remission and response (eg, using the PHQ-9 for evaluating DSM-based depressive symptoms) compared with those receiving regular in-person CBT without the addition of EMI. It would also be crucial to test the hypothesized mediating role of self-efficacy in these future studies to elucidate the mechanisms of change in the CBT-EMI.

### Conclusions

Despite the abundance of MHapps developed, the observation that none could be readily adopted to target specific clinical or therapeutic needs across settings may substantially prevent the scaling up of digitally assisted mental health care across populations. By demonstrating how no-code platforms can accelerate the development of theory-based interventions and how blended CBT-EMI approaches can effectively extend therapeutic engagement beyond clinical settings, this work presents a replicable roadmap for developing scalable, person-centered digital mental health interventions. It would be essential for future work to test the efficacy of the blended care intervention in large-scale randomized controlled trial studies.

## Supplementary material

10.2196/77036 Multimedia Appendix 1 Additional material.

## Abbreviations

BCT: behavior change technique
CBT: cognitive behavioral therapy
EMI: ecological momentary intervention
ESM: experience sampling method
HAPA: Health Action Process Approach
IDEAS: Integrate, Design, Assess, and Share
MHapp: mental health app
PHQ-9: Patient Health Questionnaire-9
TORCH: Technology provision, On-site technical support, Rehearsal, Connection with group members, and Hardcopy notes
UX: user experience
