# Supplementary material for: Leveraging No-Code Digital Platforms for Designing an Integrated Smartphone-Based Ecological Momentary Intervention With Cognitive Behavioral Therapy for Mental Health Care: Development and Usability Study
Source: JMIR Form Res. 2025 Nov 21;9:e77036. doi: 10.2196/77036 (PMC12638038; doi:10.2196/77036)
Supplement: Multimedia Appendix 1 [file formative-v9-e77036-s001.pdf]

Multimedia Appendix

Table S1. Use of different design thinking activities and objectives.. 2
Table S2. Synthesis of all MHapps identified in Bakker et al. and Linardon et al... 3
Table S3. User needs and feedback and detailed descriptions of adaptations made to the CBT-EMI.............10
Table S4. Preferences for various features in the MHapp for the blended CBT-EMI.......................................15

Table S1. Use of different design thinking activities and objectives

Activity (Session #)	Description	Objective
Empathy Map (S1)	A visualisation method to facilitate participants in identifying the thoughts, feelings, habitual behaviours, and desires of different potential service users. In the *Peer Supporters* group, this activity was conducted with regard to two personas (one with mild depressive symptoms, has good social support but missed two CBT sessions and lacks motivation in using smartphone apps; one with moderately severe depressive symptoms with high motivation in completing the CBT sessions and homework, although has a persistent feeling of bodily pain after a recent fall at home). In the *service users* group, this activity was conducted based on their personal experiences, with no persona given.	To determine the needs of individuals with different presenting problems, circumstances, and preferences by empathising with them.
Product Pinocchio[a] (S1)	A game design method, wherein the MHapp (product) is personified as a 'friend' for participants to consider what qualities and features will facilitate or be barriers to the sustained use of the MHapp for CBT-EMI homework activities.	To facilitate the identification of features deemed important in the CBT-EMI platform.
Eisenhower Matrix (S1/2)	A 2x2 matrix spanning from least to most urgent on one dimension and from least to most important on another dimension. Participants referred to the needs of potential users of the CBT-EMI MHapp, as identified during the Empathy Map activity, and positioned them accordingly on the matrix.	To determine which user needs should be prioritised in the development process of the CBT-EMI MHapp.
MHapp logistics discussion (S2/3)	An activity designed by the team, inviting participants to comment on the logistics aspects of the CBT-EMI MHapp, particularly with reference to EMI reminders: preferred number of EMI beeps per day, the time of day for the beeps, random or fixed beep reminders, and any other preferred features.	To ensure that the frequency and method of delivering CBT-EMI beeps will be acceptable to participants.
MHapp interface discussion (S2/3)	Participants were provided with printouts of various landing pages of the CBT-EMI MHapp prototype, including smartphone mock-ups and stickers of existing icons, to freely edit and rearrange components on the interface, as well as make suggestions. Additional discussions were made concerning the choice of words and illustrations used.	To allow participants to design the CBT-EMI MHapp interface directly and collaboratively.

[a] Only completed with Peer Supporters.

Table S2. Synthesis of all MHapps identified in Bakker et al. and Linardon et al.

App name	Availability	Adaptable?	Key functions	Developer	Last update[a]	Trial tested?
**iOS and Android (n = 35)**						
7 Cups	Public	x	Emotional support; Community forums	7 Cups of Tea, Co.	26 Mar 2025	✓
Body+	Public	x	Mood tracker; Guided exercises to challenge negative thoughts related to body image and build body acceptance	GGtude Ltd.	4 Oct 2022	✓
Calm	Public	✓[k]	Guided meditations; Relaxation exercises and resources	Calm.com, Inc.	29 Apr 2025	✓
COVID Coach	Public	x	Mood tracker; Guided exercises and resources	US Department of Veterans Affairs (VA)	22 Jan 2025	✓
DARE	Public	x	Guided exercise; Well-being resources	BMD Publishing Ltd.	31 Mar 2025	✓
Daylight	Restricted[h]	x	Guided exercise	Big Health Ltd.	28 Feb 2025	✓
OCD.app - Anxiety, PTSD, CBT[b]	Public	x	Mood tracker; Guided exercises and resources	GGtude Ltd.	30 Apr 2025	✓
Happier Meditation[c]	Public	x	Guided exercises	Happier Meditation, Inc.	24 Apr 2025	✓
Happify	Public	x	CBT-based games and activities; Journal	Twill Inc.	28 Jun 2024	✓
Headspace: Sleep & Meditation	Public	✓[k]	Guided exercises; AI mental health support; Scheduling of professional mental health support	Headspace Inc.	28 Apr 2025	✓
Healthy Minds Program	Public	✓[k]	Guided exercises; Resources	Healthy Minds Innovations, Inc	24 Apr 2025	✓
iMHere 2.0[d]	Restricted[i]	x	Self-management tasks; Contact with mental health clinicians	University of Pittsburgh	14 Nov 2024	✓
InMind	Public	x	Heart rate measurement; Mood diary; Guided exercises	Demand Co, Ltd.	13 Oct 2024	✓
Insomnia Coach	Public	x	Sleep diary; Guided exercises; Contact with mental health clinicians (sleep coach)	US Department of Veterans Affairs (VA)	20 Nov 2024	✓
Intellect: Create a Better You	Public	x	Contact with mental health clinicians; Mood tracker; Guided exercises	Intellect Company	12 Apr 2025	✓
IntelliCare Plus	Public	x	Mood tracker; Well-being resources (e.g., to challenge thinking, cultivate gratitude, cope with stress)	Adaptive Health, Inc.	4 Jan 2024	✓
LiveWell	Restricted[g]	x	Habit tracker; Guided resources and exercises	Zurich Insurance Group Ltd.	17 Apr 2025	✓
MCT & More	Public	x	Self-help exercises targeting different psychological problems	A scholar from the University Medical Center Hamburg-Eppendorf, Hamburg, Germany	Sept 23, 2024	✓
Meru Health	Public	x	12-week programme, including individual therapy sessions with	Meru Health, Inc.	8 Apr 2025	✓

			mental health clinicians, anonymous community support, biofeedback breathing device, and other guided exercises			
MindShift CBT - Anxiety Relief	Public	x	CBT-based exercises (daily check-in, goal setting, thought journals, belief experiments); Guided exercises; Community forum	247 Labs Inc.	13 Mar 2025	✓
Mood Mission	Public	x	Guided exercises	MoodMission Ptd Ltd.	22 Nov 2023	✓
MyPossibleSelf: Mental Health	Public	x	CBT-based in-the-moment tools and coping strategies (e.g., mood tracker, reframing negative thoughts); Guided exercises; Resources	My Possible Self Limited	1 Oct 2024	✓
PTSD Coach	Public	x	Guided exercises; Symptom tracker; Psychoeducational materials; Self-assessment	US Department of Veterans Affairs (VA)	4 Apr 2024	✓
PTSD Family Coach	Public	x	Guided exercises; Symptom tracker; Psychoeducational materials; Resources (on taking care of oneself and others); Community forum	US Department of Veterans Affairs (VA)	14 Dec 2023	✓
REM Volver a casa[e]	Public	x	Guided exercises; Resources	A clinical team from the Spanish National Health System, La Paz University Hospital, Príncipe de Asturias Hospital, and Autonomous and Alcalá Universities, Madrid	6 Aug 2024	✓
SAM (Self-help for Anxiety Management)	Public	x	Self-help techniques; Anxiety tracker; Social cloud (an anonymous social network for sharing concerns and offering/gaining support)	Mind Garden Technology C.I.C; a team at the University of the West of England	6 Mar 2024	✓
Serene: Practice Self-Care	Public	x	CBT and MBCT-based activities (e.g., guided mindfulness and self-compassion meditation, cognitive restructuring, and mindful journaling); Wellness check-in (including sleep, food, and exercise)	A clinical and counselling psychology doctoral student at the University of Toronto	10 May 2022	✓
Smiling Mind	Public	x	Resources and content to train mental fitness	Smiling Mind Pty Ltd.	1 May 2025	✓
Step-by-Step	Public	x	Self-help intervention following the “Step-by-Step” programme developed by the WHO for people residing in Lebanon	Exquitech / Inspire Solutions (by the Ministry of Public Health, Lebanon)	10 May 2024	✓
Stresscoach	Public	x	Guided exercises; Sleep-related resources	Stresscoach GmbH	21 Mar 2023	✓
StressProffen	Restricted[j]	x	Guided exercises; Resources	Department for Digital Health Research at Oslo University Hospital	7 Apr 2025	✓
SuperBetter	Public	x	Mental health challenges and games	SuperBetter, LLC.	29 Aug 2023	✓
Unwinding Anxiety	Public	x	Guided exercises; Resources	Sharecare, Inc.	17 Apr 2025	✓

VGZ Mindfulness Coach[f]	Public	x	Guided exercises	VGZ Zorgverzekeraar N.V.	5 Feb 2025	✓
What's Up? A Mental Health App	Public	x	CBT and ACT-based strategies for coping; Thoughts and mood diary; Habit tracker; Motivational quotes	Jackson Tempra	3 Oct 2024	x
**iOS-only (n = 5)**						
DBT Diary Card and Skills Coach	Public	x	Reference manual; Behaviour tracker; Skills coach	Durham DBT, Inc.	28 Oct 2019	x
EASYHEiGHTS	Public	x	Guided training via virtual reality for managing fear of heights (exposure)	Clinical research team at the University of Basel	22 Feb 2022	✓
iMoodJournal - Mood Diary	Public	x	Mood diary and mood tracker; Visualisation of moods on map (if geolocation is enabled)	Inexika Inc.	22 Mar 2025	✓
Moodhacker®	Public	x	Mood tracker; Guided exercises	GlobalMedia Group, LLC	n/a	✓
SUPPORT Coach[g]	Public	x	Symptom assessment; Guided exercises, particularly for traumatic stress symptoms	Academisch Medisch Centrum	15 Jan 2019	✓
**Android-only (n = 3)**						
Building Resilience	Public	x	Learning resources	92 Interactive Inc.	12 Jun 2023	✓
RACGP Healthy Habits	Public	✓[k]	Healthy habits tracker; Resources hub (incl. physical activity, nutrition, sleep health)	The Royal Australian College of General Practitioners	17 Mar 2025	x
Renew	Public	x	Mood tracker; CBT-based guided activities for recovery from PTSD symptoms	Palo Alto Veterans Institute for Research	17 Sep 2024	✓
**Cannot be downloaded/No longer available (n = 106)**						
AnxietyCoach	–	–	–	–	–	x
Aptivate!	–	–	–	–	–	✓
Aramgar	–	–	–	–	–	✓
BCSzone	–	–	–	–	–	✓
Be Good to Yourself	–	–	–	–	–	✓
BEAM	–	–	–	–	–	✓
Behavioral Experiments	–	–	–	–	–	x
BetterLife	–	–	–	–	–	✓
BioBase	–	–	–	–	–	✓
Boost Me	–	–	–	–	–	✓
Break Binge Eating	–	–	–	–	–	✓

Breathe	–	–	–	–	–	x
Calm in the Operating Room	–	–	–	–	–	
CARE	–	–	–	–	–	✓
CareMom	–	–	–	–	–	✓
ClinTouch	–	–	–	–	–	✓
Combating Stress	–	–	–	–	–	✓
Complex matrix	–	–	–	–	–	✓
CORE	–	–	–	–	–	✓
Depression Prevention	–	–	–	–	–	x
DeStressify	–	–	–	–	–	✓
DREAMLAND	–	–	–	–	–	✓
eMums Plus	–	–	–	–	–	✓
Feel Stress Free	–	–	–	–	–	✓
Fit Brains	–	–	–	–	–	✓
Flowy	–	–	–	–	–	✓
FOCUS	–	–	–	–	–	✓
Food ICT	–	–	–	–	–	✓
Foundations	–	–	–	–	–	✓
Get Happy	–	–	–	–	–	✓
GG – Relationship doubts and obsessions	–	–	–	–	–	✓
GG Relationship Doubts	–	–	–	–	–	✓
Happy Mom	–	–	–	–	–	✓
Happy Mother	–	–	–	–	–	✓
HARU ASD	–	–	–	–	–	✓
HARUToday	–	–	–	–	–	✓
HeadGear	–	–	–	–	–	✓
Health Minds	–	–	–	–	–	✓
HIAF	–	–	–	–	–	x
Ibobbly	–	–	–	–	–	✓
iCouch CBT	–	–	–	–	–	✓

iCounselor - iCounselor: Depression, iCounselor: Anger, iCounselor: Anxiety	–	–	–	–	–	x
iParent	–	–	–	–	–	✓
ImproveYourMood	–	–	–	–	–	✓
In Hand	–	–	–	–	–	x
Individualized	–	–	–	–	–	✓
iPST	–	–	–	–	–	✓
It’s Time to Relax	–	–	–	–	–	✓
Kaiketsu-App	–	–	–	–	–	✓
LifeBuoy	–	–	–	–	–	✓
Lifestyle Hub	–	–	–	–	–	✓
Luna Luna Baby	–	–	–	–	–	✓
MibyeongBogam	–	–	–	–	–	✓
Mindful Senses	–	–	–	–	–	✓
Mindfulness for Growth and Resilience	–	–	–	–	–	✓
Mindtastic	–	–	–	–	–	✓
Mobiletype	–	–	–	–	–	✓
Mood Kit	–	–	–	–	–	✓
Mood Prism	–	–	–	–	–	✓
Mood Tracker	–	–	–	–	–	✓
MoodKit	–	–	–	–	–	✓
Moodlytics	–	–	–	–	–	x
Moody Me	–	–	–	–	–	x
MT-Phoenix	–	–	–	–	–	✓
My Home Midwife	–	–	–	–	–	✓
NHSWBP	–	–	–	–	–	✓
Nod	–	–	–	–	–	✓
Pacifica	–	–	–	–	–	✓
Perspectives	–	–	–	–	–	✓

Pocket CBT	–	–	–	–	–	x
ProACT-S	–	–	–	–	–	✓
Project EVO	–	–	–	–	–	✓
PsyApp	–	–	–	–	–	✓
PsyCovidApp	–	–	–	–	–	✓
Refresh	–	–	–	–	–	✓
resleep	–	–	–	–	–	✓
Run4Love	–	–	–	–	–	✓
Self-guided/Therapist-guided Blueprint	–	–	–	–	–	✓
Simple matrix	–	–	–	–	–	✓
Stress & Anxiety Companion	–	–	–	–	–	x
SkillJoy	–	–	–	–	–	✓
SleepCare	–	–	–	–	–	✓
Sleep Ninja	–	–	–	–	–	✓
SMART	–	–	–	–	–	✓
Spirits Healing	–	–	–	–	–	✓
Spring	–	–	–	–	–	✓
Stop, Breathe, Think	–	–	–	–	–	✓
StressLess	–	–	–	–	–	✓
TEO m-PHA	–	–	–	–	–	✓
ThinkHappy	–	–	–	–	–	x
Thought Challenger	–	–	–	–	–	✓
Todac Todac	–	–	–	–	–	✓
Todaki	–	–	–	–	–	✓
W-GenZ	–	–	–	–	–	✓
We’ll App	–	–	–	–	–	✓
WeClick	–	–	–	–	–	✓
Whitu	–	–	–	–	–	✓
Woebot	–	–	–	–	–	✓
Working with Worry	–	–	–	–	–	✓

WorkOut	–	–	–	–	–	x
WorryTime	–	–	–	–	–	x
XiaoE	–	–	–	–	–	✓
XiaoNan	–	–	–	–	–	✓
Zemedy	–	–	–	–	–	✓
ZENN	–	–	–	–	–	✓
ZoroPhobia	–	–	–	–	–	✓
**Not a MHapp (n = 1)**						
WeChat	–	–	–	–	–	n/a

[a] For MHapps available on both iOS and Android, the date of last update is based on that indicated on the iOS App Store.

[b] *OCD.app - Anxiety, PTSD, CBT* is formerly known as *GG OCD, Anxiety and depression*.

[c] *Happier Meditation* is formerly known as *10% Happier*.

[d] *iMHere 2.0* is formerly known as *iMHere*.

[e] *REM Volver a casa* is available only in Spanish.

[f] *VGZ Mindfulness Coach* is available only in Dutch.

[g] *SUPPORT Coach* is available only in Dutch.

[h] Access to the app *Daylight* is limited to US citizens covered by certain employers/health plans and UK citizens covered by NHS projects in selected areas.

[i] Access to the apps *iMHere 2.0* and *StressProffen* is limited to participants of the developer's research study.

[j] Access to the app *LiveWell* is limited to employees of partnering corporations.

[k] The four apps (*Calm; Headspace; Healthy Minds Program; and RACGP Healthy Habits*) that can be adapted only allow customisation of existing functions and content; individual researchers or clinicians can add no new content.

Note. All MHapps reviewed are those reported in Bakker et al. [1] and Linardon et al. [2]. ACT = acceptance and commitment therapy; CBT = cognitive behavioural therapy; DBT = dialectical behaviour therapy; MCT = metacognitive therapy; PTSD = post-traumatic stress disorder; RACGP = The Royal Australian College of General Practitioners; WHO = World Health Organization.

Table S3. User needs and feedback and detailed descriptions of adaptations made to the CBT-EMI

User needs and feedback			Source	Adapted?	Descriptions of the adaptations to the CBT-EMI
**Sampling scheme**	1.	Enable customisation of the number of beeps for EMI reminders to enhance flexibility.	PS	✓	Instead of fixating on 3 beeps per day, participants agreed upon having 2 beeps per day (morning and night). If individual clients wish to complete more EMI activities, they can do so at their preferred time using the app.
	2.	Schedule 2 beeps a day, one before noon and one at night.	Both	✓	Both groups agreed to 9 a.m. and 9 p.m.
	3.	Enable flexibility in responding to the EMI beeps, such that participants could fill in the questionnaire later after the beep time.	Both	✓	Rather than requiring a short period of responding to EMI beeps (e.g., within 15 minutes, we extended the time for responding to a beep to 3 hours, whilst encouraging participants to complete the beeps as soon as they received them.
	4.	Require the completion of a set number of EMI activities (12 in total, without specification of time or day of completion) in the subsequent six days before the next CBT session rather than completing 2 EMI beeps per day.	PS	x	We did not make this adaptation because (1) service users expressed acceptability in receiving 2 beeps daily, and (2) from the clinical and research perspective, the use of EMI was targeted at facilitating participants to enact CBT-based activities across settings and contexts, as well as at differing emotional states (tackling the BCT "Behavioural practice/rehearsal").
	5.	Enable participants to mute the beeps if needed.	PS	x	We did not make adaptations because individual participants could implement this via their smartphone settings. Service users also thought adding the muting function was not needed, as the activities were not 'mandatory' (i.e., without consequence) and participants could choose flexibly whether or not to respond to the beeps.
	6.	Implement only Mood Check across all beeps to shorten the overall EMI completion time.	PS	✓	Mood Check was prompted at every beep, with additional CBT-based activities prompted after the first week according to the intervention schedule. This ensures a shorter EMI completion time (increases motivation to complete), whilst facilitating participants to develop a habit of being aware of their mood states across settings.
**Question types**	7.	Insufficient options in the multiple choice questions (e.g., relaxation exercises, activity logging); add an 'Other' and a free-text response option for participants who want to document their experiences in greater detail.	PS	✓	A wider range of relaxation exercises was provided. For triggering events and activity logging questions, additional examples were provided to ensure a more comprehensive range of experiences can be captured, with an 'Other' option and free-text response option provided.
	8.	Rearrange the question flow, such that questions likely to prime negative emotions are placed at the beginning.	PS	✓	We reviewed our question types and confirmed that questions likely to prime negative emotions (e.g., logging a negative event) were placed at the beginning.
	9.	Provide suggested statements for open-text questions as prompts	PS	n/a	*Non-adaptable by individual researchers/clinicians on m-Path.*

	for responses to reduce the level of difficulty.			
	10. Adopt the photo-upload question type ('Plus Point'), which appeared interesting, although the term should be amended as it is slightly confusing in the present context.	PS	✓	We incorporated the 'Plus Point' feature into the CBT-EMI, allowing participants to complete the 'Gratitude Diary' activity. Participants can log any event or person they were grateful for on the day of the beep with a photograph as a supplement. All these logged entries can be viewed at any time. New diary entries could also be logged at any preferred time. For the present intervention context, the term 'Plus Point' was updated to 'Diary' in traditional Chinese.
	11. Adopt briefer question types (e.g., multiple choice questions).	SU	✓	We reviewed our question types and incorporated more multiple choice questions.
	12. Allow users to select multiple options in multiple choice questions to enhance their functionality.	SU	✓	We enabled the multiple selection setting for multiple choice questions.
**App design and user experience**	13. Make the questions and prompts more straightforward (e.g., questions to be more specific rather than generic).	SU	✓	We refined all questions to ensure their brevity based on suggestions made by the service users. Some activities with more generic phrases (e.g., 'distress' in the *Wellness Toolbox* activity rather than 'sad' or 'irritated') were retained to ensure their relevance to a wider range of potential users and across varied mood states. Nevertheless, all activities are anticipated to be demonstrated and practised with the practitioner during the actual CBT sessions to ensure their personal relevance to individual participants.
	14. Use figures and icons that are more age-inclusive and appear 'old'.	PS	✓	We re-designed the figures and human icons featured in the MHapp, edited the text to use more age-friendly language, and tested it with service users, who found it acceptable (**Figure 4**).
	15. Develop two interfaces, one for young-old users and another for old-old users.	PS	x	With the refined figures used (**Figure 4**), the MHapp was deemed suitable even for young-olds. The overall design was also deemed appropriate by service users, hence we considered it sufficient to design one simple-to-use MHapp for all older adults.
	16. Incorporate a 'daily check-in' element with encouraging messages (e.g., on home screen) to motivate participants to develop a habit of completing exercises on the MHapp.	PS	x	A 'home button' can be added by individual researchers/clinicians to the home screen of the MHapp, which can lead users to specific questions/activities. While the text in the home button can be customised, it is not yet possible to automate the revelation of new texts to be shown periodically. Nevertheless, we amended the reminder message of the EMI beeps to be more user-friendly to create a more welcoming tone to encourage use.
	17. Customise the reminder messages to make them more engaging and attention-grabbing so that participants will notice and respond.	PS	✓	We edited the beep reminder message to be more inviting and engaging (e.g., "Morning 🍃Time to relax a little!").

	18. Enable users to refine their answers to previous questions in the same beep.	Both	✓	We enabled the 'Go-Back' function.
	19. Incorporate a clearly labelled 'back' button for users to move through the MHapp.	PS	n/a	*Non-adaptable by individual researchers/clinicians on m-Path.*
	20. Increase button sizes within the app to accommodate smartphone screens with lower sensitivity, making it easier for users.	PS	n/a	*Non-adaptable by individual researchers/clinicians on m-Path. (However, font sizes can be increased by adjusting individual smartphones' settings.)*
	21. Add a magnifying function for participants when clicking on an image.	PS	n/a	*Non-adaptable by individual researchers/clinicians on m-Path.*
	22. Implement more customised profile settings by allowing users to upload profile pictures.	PS	n/a	*Non-adaptable by individual researchers/clinicians on m-Path.*
	23. Add a button popping up at the front page that activates upon completion of exercises (e.g. Daily Challenge). Notify participants when exercises are completed or pending.	PS	n/a	*Non-adaptable by individual researchers/clinicians on m-Path.*
	24. Visualise upcoming scheduled exercises to keep users engaged and motivated, preferably in a calendar view.	PS	n/a	*Non-adaptable by individual researchers/clinicians on m-Path.*
	25. Incorporate a 'prize' function with a 'congratulatory sound' upon completion of an exercise.	PS	✓	We activated the virtual badge as 'awards' on the MHapp to increase motivation and a degree of gamification. There is, however, no congratulatory sound available.
	26. Highlight that the information is from credible sources when presenting materials and exercises to address concerns about fake news and scams online.	Both	✓	Reassurance of credibility was addressed through: (1) explanation of each activity during the in-person CBT sessions, and (2) adding an optional "know more about this activity" section to explain the rationale and research background of each activity.
**Homework-specific:** ***Mood Check***	27. Figures that indicate emotions should be more explicit, such as using more obvious signs for an angry face.	PS	✓	We redesigned the figures and human icons featured in the MHapp.
	28. Provide more explanation on what each of the emotions means (e.g., what is meant by 'anxiety'), as simply selecting a score cannot help the user reflect on why those emotions occur.	PS	✓	We reviewed and confirmed that these items will be discussed during the CBT sessions, which will provide users with a foundation to understand and reflect upon the different mood states. The activity will also be demonstrated, rehearsed, and discussed in sessions.
	29. Even though labels are provided below the anchors (1 and 7), it was unclear what the low and high scores refer to, making it difficult to accurately reflect one's mood states.	SU	✓	We added more detail directly in each Mood Check item to facilitate understanding (i.e., "1 point = none, 7 points = very"). These should also be clearer in the actual intervention as each item will be demonstrated, rehearsed, and discussed in the CBT sessions.
	30. Asking about mood states within a 1-hour timeframe may be too short, as events that occurred earlier could still impact a person's mood states throughout the day or even across multiple days.	SU	✓	We referenced prior ESM and EMI work in the field, reconsidered the rationale behind the activity, and reached a consensus that the activity was intended to prompt users to reflect on their mood

				states at the time of completion. The questions therefore focus on asking about users' mood states at the present moment ("right now").
Behavioural Activation	31. Incorporate additional multimedia elements, such as short videos (e.g., guided mindfulness videos) with regular updates.	Both	✓	We added images throughout the exercises, GIFs and videos at the end of each beep, and additional guided relaxation exercises and behavioural activation videos.
	32. Incorporate a wide range of easy-to-digest reading materials within the MHapp, allowing users to read at their own convenience.	Both	✓	We condensed and added the materials taught in the in-person sessions (e.g., functions of different emotions, sleep hygiene) to the 'home button', allowing users to access them at any time beyond the pre-set EMI beeps.
'Hot Cross Bun' and Gratitude Diary	33. Provide more space to describe the triggering events (lack of space for writing in the MHapp prototype).	SU	✓	We initially intended to reduce user burden and thus opted to provide multiple choice options for logging their recent experience in the Hot Cross Bun activity. This was, nonetheless, considered to be too restricted and limits what users can express. We therefore amended the question type to open text response. In addition, for those who wish to log their experiences beyond the EMI beep, users can freely use the 'Plus Point' function (or 'Diary' in Chinese) to avoid lengthening the overall activity.
	34. Requiring the documentation of 1 thing/person to be grateful for instead of 3 would be more manageable. Briefly recording it on some days across a week, rather than demanding a detailed account on a specific day, will be preferable.	SU	✓	While it is encouraged that users record 3 things/people they are grateful for, we amended the activity based on users' feedback, namely to encourage rather than require documenting 3 things/people they are grateful for and prompted this once (together with the EMI beep in the evening) over the week rather than daily. This was considered more important in increasing motivation among users to use the MHapp.
**Feedback beyond the MHapp**	35. Instead of beeping users using the built-in reminder from the system, SMS messages or WhatsApp reminders could be incorporated to increase the completion rate.	Both	✓	Will be incorporated into the protocol of the CBT-EMI.
	36. Provide a contact point for initial demonstration of the app and ongoing troubleshooting.	Both	✓	A manual and intervention protocol will be developed to facilitate future service providers in the implementation of the CBT-EMI (including those who may be less familiar with *m-Path*). A member of the current research team will provide support to any interested service provider at the initial stage of its implementation; further training will be developed to improve its scalability with other means of support provided (e.g., setting up a WhatsApp group for enquiries, a regularly updated FAQ page).
	37. Incorporate additional human support (e.g., from Peer Supporters) to guide users in using the app and check-ups for those who did not respond (e.g., after 2 full days, i.e., 4 consecutive beeps).	Both	/	Additional feasibility testing of the *m-Path* will be made (e.g., setting up a system to notify Peer Supporters upon non-response from users for 2 days) before adopting this suggestion, with

			consideration of the potential burden on Peer Supporters. Upon adoption of this suggestion, a manual will be co-developed with Peer Supporters to clarify their roles in the process (including potential risks, boundaries, and self-care).
38. Match accounts to family members or peer supporters so that they can check users' records regularly.	PS	/	Will be reconsidered in strategic planning at a later stage, with reference to potential privacy concerns and individual users' preferences.
39. Make the app more multi-functional (instead of a stand-alone app just for mental health), including a function to connect with others.	PS	/	Will be considered in strategic planning at a later stage (i.e., setting up a WhatsApp group for communication and encouragement).
40. Offer tangible incentives beyond the MHapp, such as financial incentives, to increase their motivation.	PS	/	Will be reconsidered in strategic planning at a later stage, with reference to potential pragmatic concerns.

Note. '✓' represents that the MHapp has been adapted based on specific user feedback and needs; 'x' represents that no adaptation after reconsiderations of the feedback; 'n/a' represents that adaptations could not be made by individual researchers/clinicians (e.g., changes only possible by the developers, namely *m-Path* team in this context), '/' represent that the feedback cannot yet be incorporated in the current stage due to manpower and resource considerations but may be considered at a later stage and by other research teams.

Table S4. Preferences for various features in the MHapp for the blended CBT-EMI

	Mean (SD) (range = 1–7)	
	Peer Supporters (n = 8)	Service users (n = 5)
**Features (1 = strongly disagree, 7 = strongly agree)**		
Multiple choice questions: allow only one answer	3.13 (2.53)	3.75 (1.26)
Multiple choice questions: allow multiple answers	6.00 (1.41)	4.75 (1.50)
Provide an 'other' option and enable self-learning in multiple choice questions[a]	4.88 (2.23)	6.50 (1.00)
Order question (respond by rearranging a set of given options in preferred order in response to a set question)	4.88 (2.36)	4.00 (2.45)
Multi-smiley question (select one or more of six possible pre-set mood states in response to a set question: *Sadness, Happiness, Anger, Fear, Stress,* and *Surprise*)	5.63 (2.13)	3.75 (2.06)
Body parts (select one or more body parts in response to a set question)	6.13 (1.46)	5.00 (2.83)
Image choice	6.50 (0.76)	5.00 (2.71)
Open text	6.43 (1.13)	5.25 (1.50)
Smiley	5.50 (1.93)	6.00 (1.15)
Slider question	6.13 (1.13)	5.50 (1.29)
Setting minimum and maximum values in slider questions	5.86 (1.35)	5.00 (1.00)

[a] Self-learning feature in m-Path: When a user adds a new response using the 'Other' option, the same response will be made available as an additional option when the user is given the same question in subsequent beeps.

Note. Data is missing from 1 service user across all items; for the items on 'open text' and 'setting minimum and maximum values', data is missing from 1 Peer Supporter.

References

1. Bakker D, Kazantzis N, Rickwood D, Rickard N. Mental health smartphone apps: Review and evidence-based recommendations for future developments. JMIR Ment Health. 2016;3(1):e7.

2. Linardon J, Torous J, Firth J, Cuijpers P, Messer M, Fuller-Tyszkiewicz M. Current evidence on the efficacy of mental health smartphone apps for symptoms of depression and anxiety. A meta-analysis of 176 randomized controlled trials. World Psychiatry. 2024;23(1):139–49.
